# Use of cellulose microfibers from olive pomace to reinforce green composites for sustainable packaging applications

**DOI:** 10.1002/fsn3.3469

**Published:** 2023-06-01

**Authors:** Cyrine Amara, Ayoub El Mahdi, Perihan Kubra Akman, Raouf Medimagh, Fatih Tornuk, Khaoula Khwaldia

**Affiliations:** ^1^ Laboratoire des Substances Naturelles, Institut National de Recherche et d'Analyse Physico‐chimique (INRAP) BiotechPole Sidi Thabet Ariana Tunisia; ^2^ Higher Institute of Biotechnology of Sidi Thabet (ISBST) University of Manouba Ariana Tunisia; ^3^ Food Engineering Department, Chemical and Metallurgical Engineering Faculty Yildiz Technical University, Davutpasa Campus Esenler, Istanbul Turkey

**Keywords:** cellulose microfibers, composite films, olive pomace, packaging

## Abstract

To valorize abundant, unexploited, and low‐cost agro‐industrial by‐products, olive pomace is proposed as a sustainable and renewable raw material for cellulose microfibers (CMFs) production. In this study, CMFs were extracted from olive pomace using alkaline and bleaching treatments and characterized in terms of morphological, structural, and thermal properties. Afterward, the reinforcing capability of microfibers was examined using carboxymethyl cellulose (CMC) as a polymer matrix by the solvent casting process. The effects of CMF loading (1%, 3%, 5%, and 10%) on the composites' mechanical, physical, morphological, and thermal properties were assessed. CMF incorporation led to a decrease in moisture content (MC), water solubility (WS), and water vapor permeability (WVP) and an increase in tensile strength (TS), stiffness and transparency values, and thermal stability of CMC films. Increasing CMF content to 5%, increased the TS and elasticity modulus by 54% and 79%, respectively, and reduced the WVP and light transmissivity at 280 nm, by 22% and 47%, respectively. The highest water, moisture, light barrier, and mechanical properties of composites were reached at 5% CMFs.

## Graphical abstract

Cellulose microfibers (CMFs) were extracted from olive pomace and their reinforcing capability was examined using carboxymethyl cellulose (CMC) as a polymer matrix. The incorporation of CMFs reduced water trapping ability and enhanced CMC films' mechanical, thermal, and water vapor barrier properties.

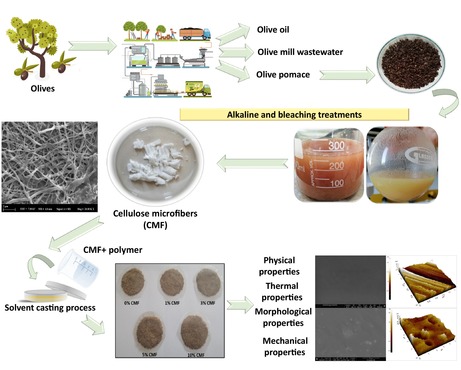

## 1 INTRODUCTION

The global production of synthetic plastics increased by approximately 37% from 2010 to 2020 and is estimated to reach 590 million metric tons by 2050 (Tiseo, 2022). However, less than 10% of all these plastics have been recycled and a great percentage of the remaining plastics has been accumulated in nature, causing disastrous environmental issues due to their non‐degradable nature, and the exhaustion of fossil fuel resources and polymers from petroleum sources (Epps et al., 2021). Substituting conventional synthetic plastics with bioplastics, derived from biobased polymers, may be an attractive solution to answer the problems mentioned above. According to European Bioplastics (2021), the total global bioplastics production amounts to 2.4 million tons in 2021 and is expected to increase by more than three times in 2026.

In this context, the use of biopolymers from biomass sources (plants, animals, and microorganisms) for the manufacture of food packaging has many advantages thanks to their outstanding properties such as biodegradability, nontoxicity, biocompatibility, and film‐forming properties. Among many bio‐based polymers, carboxymethyl cellulose (CMC), a low‐cost anionic polysaccharide, has been successfully used to produce biodegradable packaging films (Bahrami & Fattahi, 2021; Fernández‐Santos et al., 2022; Oun & Rhim, 2019) and coatings on paper packaging materials (Basta et al., 2015; He et al., 2020) with excellent optical and gas barrier properties. However, their resulting low mechanical and water vapor barrier properties limit their practical use for food packaging applications. Developing CMC‐based composite films by incorporating biopolymers (Salama et al., 2019), lipids (Mei et al., 2021), nanoclay (He et al., 2019), chitin nanocrystals (Oun & Rhim, 2019), and nanocellulose (He et al., 2020; Oun & Rhim, 2016) have been proposed as an effective strategy to overcome these drawbacks.

Nowadays, agriculture co‐ and by‐products are regarded as an available and cheap source of valuable and renewable residues including biopolymers, nanoparticles, inorganic compounds, fibers, and bioactive agents. The recovery of these added‐value residues and their exploitation in food packaging for the enhancement of the functionality of biodegradable films and coatings constitute a sustainable strategy to create value from waste, restrain the disastrous waste disposal issues, and contribute to the development of a circular economy. In this context, olive pomace, a by‐product from olive oil processing mills, accounts for more than 35% of the total weight of processed olives (Akay et al., 2015) and contains several high‐added value compounds such as lignocellulosic materials, polyphenols, lipids, and uronic acids (Khwaldia et al., 2022).

Cellulose is a fascinating biopolymer known for its good structural and functional properties, low cost, biodegradability, biocompatibility, and availability (Ejaz et al., 2020; Hamed et al., 2012; Hassan et al., 2014). Its use as a green reinforcing agent in polymer composites has been reported in many studies (Freitas et al., 2022; Hassan & Fowler, 2022) as a potential alternative to petrochemical‐based materials, and this biopolymer may find application in food, packaging, agriculture, medical, automotive, and aeronautics industries (Linan et al., 2021). It is worth mentioning that the possibility of isolating cellulose from agro‐industrial by‐products such as olive pomace has increased its popularity and uses. The conversion of low‐value agro‐industrial by‐products into useful biopolymers offers several advantages including reduction of resource use and waste generation, reduction of feedstock costs, contribution to mitigating environmental concerns related to the great dependence on products derived from petrochemicals and greenhouse gas emissions, preservation of global biodiversity and sustainable land use, and generation of new market opportunities for rural smallholder producers (Khwaldia et al., 2022; Linan et al., 2021; Patel & Shah, 2021). Moreover, this may contribute to the implementation of the principles of sustainable development and circular bioeconomy and the promotion of the zero‐waste concept to meet the new consumers' needs.

Upon processing, cellulose microfibers (CMFs), a form of nanocellulose, can be obtained with promising features such as high elastic modulus, high aspect ratio, high tensile strength, high specific surface area, low density, biodegradability, and low energy consumption (Solikhin et al., 2018; Tian et al., 2020). Cellulose fibers have been successfully applied in active packaging materials, intelligent packaging, and composite materials (Wang et al., 2022). The incorporation of CMFs in composites aims at improving packaging properties in terms of mechanical, functional, and thermal properties, thus offering the highest preservation degree of the quality and safety of foods (Amara et al., 2021). Some studies in the literature have processed composites with CMFs from different sources, such as poly (vinyl acetate)‐CMFs from eucalyptus‐bleached kraft pulp (Nozaki & Lona, 2021), thermoplastic starch‐CMFs from rice straw (Freitas et al., 2021), and polyhydroxyalkanoate and polylactic acid (PLA)‐CMFs from kraft paper (Mármol et al., 2020). Starch‐based biocomposites incorporating cellulose fibers of different sizes have been produced and exhibited a wide range of physicochemical, thermal, optical, and mechanical properties (Khalili et al., 2023). At 20 wt% of CMFs isolated from alfa fibers, the stiffness of starch‐based composites increased by 300% (Khalili et al., 2023). The incorporation of CMFs isolated from cotton noil enhanced the UV light absorbance and water vapor barrier properties of PLA‐based composites due to the uniform dispersion of CMFs in the PLA matrix and their good interfacial adhesion (Ponnusamy et al., 2022).

To the best of our knowledge, only two papers have reported the isolation of cellulose and cellulose nanocrystals (CNC) from olive pomace and olive stones, respectively (Hamed et al., 2012; Hassan et al., 2014). However, the isolation of CMFs from olive by‐products and the exploitation of their reinforcement effect to process biodegradable composites have not yet been reported. Therefore, in the present study, CMFs were isolated from olive pomace and added to CMC‐based formulations to enhance the performance of the resulting green composites. The objective of our study was to assess the effect of incorporating increasing amounts of CMFs on the barrier, mechanical, thermal, and optical properties of the processed CMC/CMF composites.

## 2 MATERIALS AND METHODS

### 2.1 Materials

Olive pomace used for cellulose extraction was kindly supplied by an olive oil mill at Sabalet Ben Ammar in the north of Tunisia during the oil‐harvesting season of 2020. Carboxymethyl cellulose (CMC) (MW ~250,000, degree of substitution 0.9, and viscosity 1500–3000 mPa.s), ethyl acetate (≥99.5%), sodium hydroxide (NaOH) (MW = 40 g/mol), sodium hypochlorite (NaClO_2_) (80% and MW = 90.44 g/mol), sulfuric acid (95%–97% and MW = 98.08 g/mol), hydrogen peroxide (30% and MW = 34.01), acetic acid (99% and MW = 60.05), and glycerol (99% and MW = 92.09 g/mol) were acquired from Sigma‐Aldrich. Peracetic acid (PAA 13%) is used as a bleaching agent and was prepared according to the methodology described in the patent of Crommelynck et al. (1981) with some modifications. The determination of PAA content was performed by the potentiometric titration using a Metrohm 785 DMP titrator.

### 2.2 Preparation of CMFs from olive pomace

**FIGURE 1:**
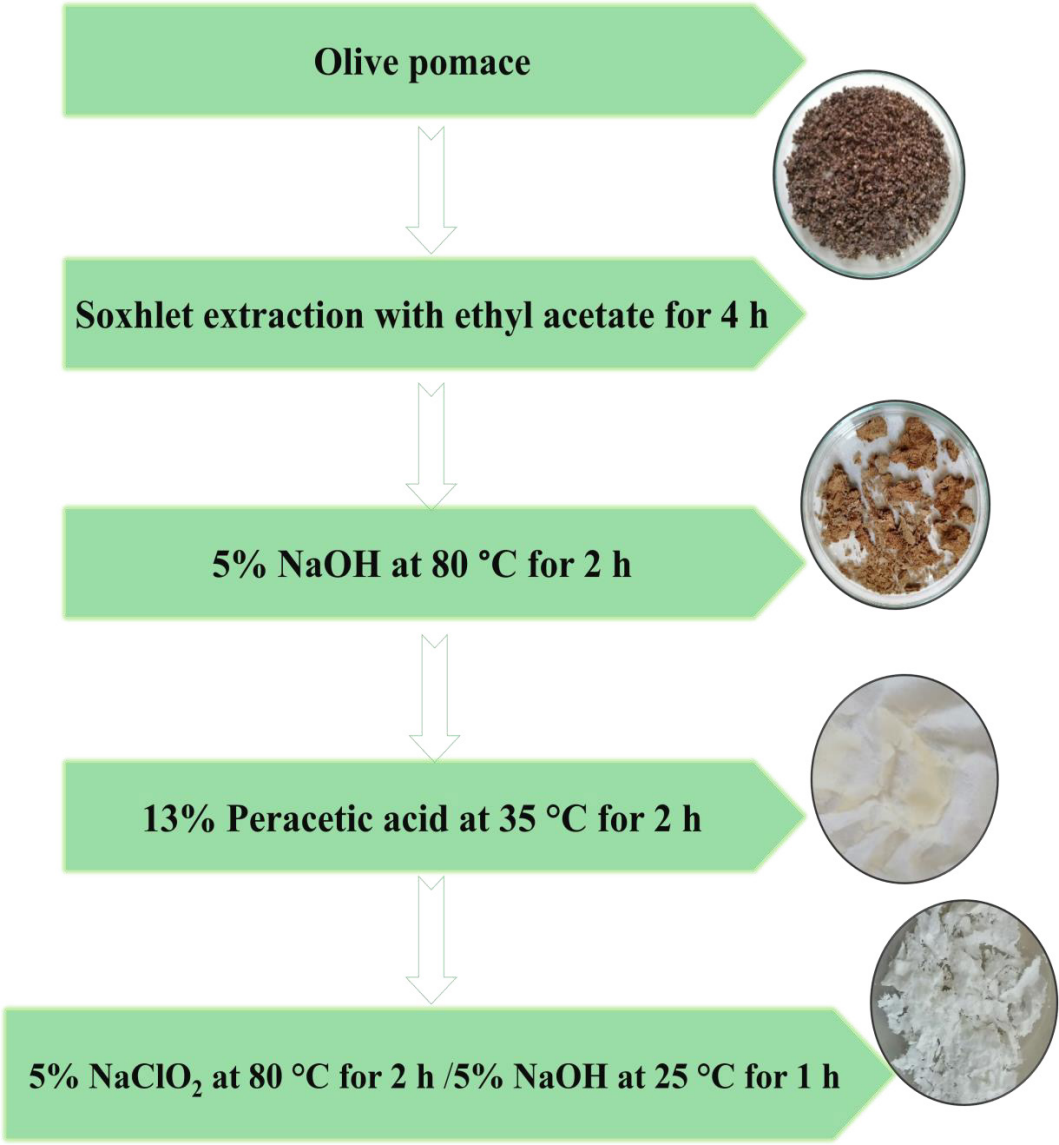
Procedure for preparing CMFs from olive pomace.

Firstly, olive pomace was subjected to Soxhlet extraction with ethyl acetate for 4 h to remove the residual materials, followed by lyophilization and crushing steps, and then further processed to obtain pure cellulose fibers. Briefly, the resulting lyophilized olive pomace was treated with an aqueous 5% NaOH solution at 80°C for 2 h, and the resulting material was bleached after filtration using 13% peracetic acid at 35°C for 2 h and then treated using, respectively, 5% NaClO_2_ at 80°C for 2 h and 5% NaOH at 25°C for 1 h. Finally, the CMF suspension was filtrated and dried at 75°C for 2 h (Figure 1). The yield of CMFs based on the initial weight of olive pomace was calculated as follows:

(1) $$\text{Yield}\;\left(\%\right)=\frac{W\;\text{Dried}\;\mathrm{CMF}}{W\;\text{Dried pomace}}\times 100$$

### 2.3 Preparation of CMC‐based films incorporating CMFs

The CMC‐CMF films were prepared using the solvent casting technique according to the methodology described by Zhang et al. (2021) with some modifications. Briefly, 2% of aqueous CMC solution (w/w) was prepared at 70°C using a magnetic stirrer for 20 min. The glycerol as a plasticizer was added to the film‐forming solutions at a concentration of 30% (w/w CMC). After incorporation of CMFs into the CMC‐based film‐forming solutions at different concentrations (1%, 3%, 5%, and 10% w/w based on CMC), the mixture was left to the continuous stirring until obtaining a homogeneous dispersion and then treated with Ultra‐Turrax (IKA T25, Digital Homogenizer) for 30 min. Finally, the resulting film solutions were cast onto Petri dishes and dried in an oven for 24 h at 45°C. The dried composite films were conditioned at 25°C and 50% RH for 2 days before testing.

### 2.4 Characterization of CMFs

#### 2.4.1 Fourier transform infrared spectroscopy (FTIR)

Fourier transform infrared spectra of CMF sample were determined by FTIR Equinox 55 spectrometer (Bruker Co.) equipped with attenuated total reflection (ATR) using diamond crystal. ATR‐FTIR spectra were performed at 4 cm^−1^ resolution and recorded between 4000 and 500 cm^−1^.

#### 2.4.2 Surface morphology analysis

The surface morphology of CMFs was observed using a scanning electron microscope (SEM, Zeiss EVO® LS 10, Carl Zeiss) operated at 7 kV under vacuum conditions.

#### 2.4.3 X‐ray diffraction (XRD)

X‐ray diffraction patterns were earned for CMF samples at room temperature using PANalytical X'Pert PRO X‐ray diffractometer using Cu‐Kα radiation (*λ* = 1.54 Å) in the range of 2*θ* from 5 to 60° at a scan rate of 5°/min. The tube current was 40 mA and the operating voltage was 45 kV. The crystallinity index (CrI) was calculated using the method of Segal et al. (1959):

(2) $$\mathrm{CrI}\;\left(\%\right)=\frac{I_{\max}-I_{\min}}{I_{\max}}\times 100$$

where *I*_max_ and *I*_min_ are the maximum and minimum intensity of the crystalline peak at 2*θ* angle between 22° and 24° and about 18°, respectively.

#### 2.4.4 Thermogravimetric analysis (TGA)

Thermogravimetric analysis (TGA) analysis of CMFs (∼10 mg) was carried out under a nitrogen atmosphere from 30 to 700°C at a heating rate of 10°C/min and a gas flow rate of 90 mL/min using thermogravimetric analyzer apparatus (TGA550, TA Instruments). The derivative thermogravimetric (DTG) curve was determined to portray the weight loss rate as a function of time with respect to the temperature.

### 2.5 Characterization of CMC/CMF composite films

#### 2.5.1 Fourier transform infrared spectroscopy (FTIR)

Fourier transform infrared spectra of CMC/CMF composite film samples were determined by FTIR as previously described.

#### 2.5.2 Surface morphology analysis

The surface morphology of CMC/CMFs was observed using a scanning electron microscope (SEM, FEI Quanta 200). Samples were fractured in liquid nitrogen and then coated with gold “Sputter coater S150” under vacuum before observation.

#### 2.5.3 Thickness

The thickness of films, performed in quadruplicate with 10 measurements taken on each film, was measured with a manual micrometer (Mitutoyo), and an average value was calculated.

#### 2.5.4 Water barrier properties

The moisture content (MC) and water solubility (WS) of films were determined using the method given by Jannatyha et al. (2020) with slight modifications. Film samples were cut into squares 2 cm × 2 cm, weighted, and dried at 105°C for 24 h to achieve a constant weight. The MC was determined by using the following equation:

(3) $$\mathrm{MC}\;\left(\%\right)=\frac{W\;\text{sample weight}-W\;\mathrm{dry}\;\text{sample weight}}{W\;\text{sample weight}\;}\times 100$$

To determine WS, dried films were immersed in 50 mL of deionized water for 12 h at room temperature. The films were filtered, and the filter paper was dried at 105°C to achieve a constant weight. The WS was calculated as follows:

(4) $$\mathrm{WS}\;\left(\%\right)=\frac{W_{1}-W_{2}}{W_{1}\;}\times 100$$

where *W*_1_ and *W*_2_ are the initial dry weight and final dry weight, respectively.

#### 2.5.5 Water vapor barrier properties

Water vapor permeability (WVP) was determined in triplicate using the gravimetric method as described in ASTM standard method E96/E96M (2015). CMC films were sealed on the top of permeation cells containing silica gel and the cells were placed in a controlled temperature (38°C) and RH (90%) chamber, and WVP was calculated as described by Gheribi et al. (2018).

#### 2.5.6 Mechanical properties

Mechanical properties in terms of tensile strength (TS), elongation at break (E), and Young's modulus (YM) of the CMC composite films were measured according to the ISO 1924‐2‐1994 method as previously described by Gheribi et al. (2018) using Instron Universal Testing Machine (Model 3345). Ten rectangular specimens of 15‐mm‐wide and 100‐mm‐long strips were cut from each film and the measurements were performed at room temperature with a separation of 100 mm and a crosshead speed of 20 mm/min.

#### 2.5.7 Optical properties

The optical transparency of the resulting films was evaluated by absorption measurements using the JASCO 630 UV–vis spectrophotometer in the wavelength range 200–800 nm. The transparency of each film formulation was calculated using the following equation:

(5) $$\text{Transparency}=-\frac{\log\left(X\right)}{T}$$

where *X* is the transmittance at 600 nm and *T* is the film thickness (mm).

A Konica Minolta SpectraMagic NX Lite CM‐S100w was used to investigate the color parameters, including L* (lightness), chroma a* (red–green), and chroma b* (yellow–blue). The measurements were conducted seven times for each film sample.

#### 2.5.8 Thermal analysis

The thermal properties of CMC‐based composite films were evaluated by differential scanning calorimetry (DSC‐60A plus, Shimadzu) with two heating scans from 30 to 300°C at a heating rate of 10°C/min under a nitrogen atmosphere.

#### 2.5.9 Statistical analysis

A completely randomized design was used by different CMF concentrations of 0%, 1%, 3%, 5%, and 10% (w/w based on CMC) to assess their effect on MC, WS, WVP, TS, %*E*, YM, color parameters, and transparency of the processed CMC/CMF composites. All results relative to film properties were reported as mean ± standard deviation (SD). One‐way ANOVA with Tukey's HSD (honestly significant difference) post hoc test was used to assess significant differences (<0.05) among studied film properties. Data were analyzed using SPSS (SPSS Inc.).

## 3 RESULTS AND DISCUSSION

### 3.1 Characterization of CMFs

**FIGURE 2:**
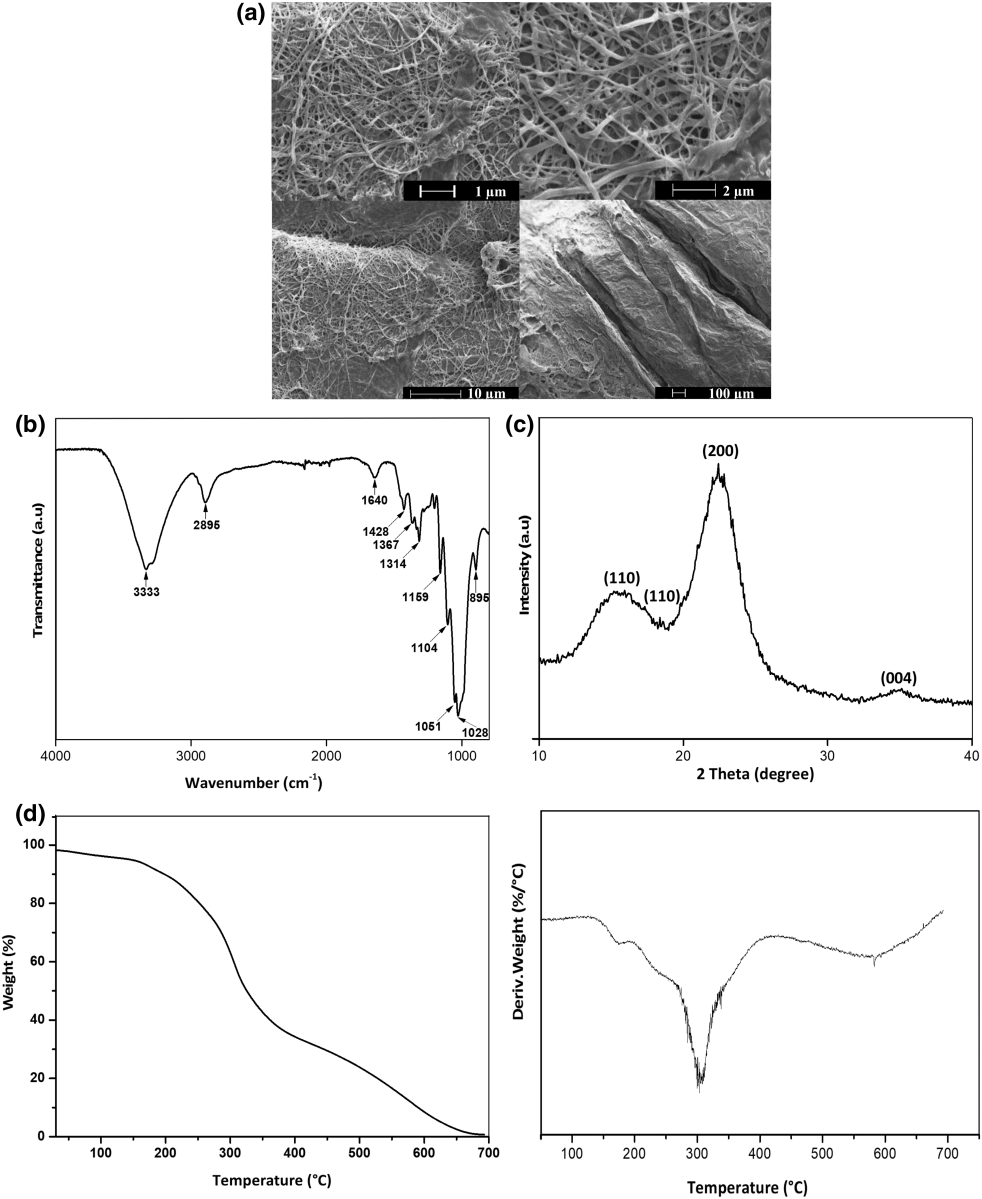
SEM images (a); FTIR (b); XRD (c); and TGA and DTG (d) spectra of CMFs derived from olive pomace.

The yield of CMFs derived from olive pomace using alkali and bleaching treatments to purify microfibers and remove hemicellulose, lignin, and extractives was 57.63 ± 0.38%. According to Khenblouche et al. (2019), the yield of CMFs extracted from *Retama raetam* was 52.1%. In another study, Reddy et al. (2016) extracted the CMFs from Palmyra palm fruit fibers. The extraction yield was 55% of the dry fibers' weight. The obtained CMF yield in our study is higher than those reported in the literature for cellulose extracted from different natural sources and this may be explained by the followed extraction process and the nature and chemical composition of raw materials used for cellulose extraction. High cellulose yields were reported in agricultural raw materials subjected to alkaline‐assisted extraction after removing lignin and hemicellulose (Leão et al., 2017). This high yield underlines the high potential of olive pomace biomass as a source of CMF thanks to its availability and low cost. To investigate the chemical structure of isolated microfibers, their functional groups were identified by FTIR measurement. The ATR‐FTIR spectrum of CMFs is illustrated in Figure 2b and allows the identification of a sharp intense bond at 3333 cm^−1^ associated with O–H stretching and intramolecular and intermolecular hydrogen bonds in cellulosic materials. The absorption peak at 2895 cm^−1^ was assigned to C–H groups and CH_2_ stretching vibration. The bond at 1640 cm^−1^ was related to the H–O–H bending vibration of absorbed water. The peak detected at 1428 cm^−1^ was assigned to –CH_2_ stretching vibration and to saccharide molecule. The characteristic peaks at 1367, 1314, and 1159 cm^−1^ were related to C–H bending regions, C–O stretching vibration of the crystalline structure of cellulose, and C–OH stretching bonds, respectively. The C–O–C symmetrical stretching vibration shows two intense peaks at 1159 and 1104 cm^−1^ indicating the pyranose ring stretching. Other peaks corresponding to C–O stretching vibration were identified at 1051 and 1028 cm^−1^. The bond at 895 cm^−1^ was ascribed to C–O–C stretching vibration of β‐glycosidic linkages between glucose in cellulose (El Achaby et al., 2018; Kassab et al., 2020). The morphological properties of CMFs isolated from olive pomace were analyzed by SEM as depicted in Figure 2a. The extracted cellulose microfibers were characterized by filamentous skeletal rod‐like structures. The micrographs showed that the cellulose fibers produced long‐shaped morphology with microfibril structures with identifiable lengths indicating the removal of hemicellulose and lignin during the chemical treatments. The average diameter of CMFs was found to be 12.27 ± 0.90 μm. Similar results were also observed in the SEM images obtained by Kassab et al. (2020), who found that the CMFs derived from hemp stalks have a diameter of 16.96 ± 1.33 μm. In another study, El Achaby et al. (2018) reported an average diameter value of 10 μm for CMFs derived from Alfa fibers using alkali and bleaching treatments. The crystalline structure of CMFs derived from olive pomace was determined by XRD analysis (Figure 2c). The obtained results exhibited value peaks at 2*θ* = 18.83° which is characteristic of the cellulose amorphous phase, while the peaks at 16.28° (110), 15.6° (110), 22.48° (200), and 34.37° (004) are attributed to the typical cellulose I structure (Reddy et al., 2018). The crystallinity index (CrI) of CMFs was measured using the Segal equation and found to be 51.81%. This obtained CrI value is higher than that of CMFs extracted from *Opuntia ficus‐indica* without any bleaching treatment (43%) (Benhamou et al., 2021; Mannai et al., 2018), suggesting the efficacy of PAA used in this study as a bleaching agent in removing the amorphous parts of non‐cellulosic components (lignin and hemicelluloses) in olive pomace, as confirmed by FTIR analysis showing the complete removal of lignin and hemicelluloses molecules. However, the CrI value is lower than that of cellulose fibers extracted from olive tree pruning biomass obtained by TEMPO‐mediated oxidation (60.26%) (Sánchez‐Gutiérrez et al., 2020). It is worth noting that this oxidation treatment maintains the crystalline structure of olive tree pruning pulp by degrading only the disordered or amorphous regions (hemicellulose) (Sánchez‐Gutiérrez et al., 2020).

The thermal degradation behavior of CMFs derived from pomace was investigated using TGA/DTG analysis as shown in Figure 2d. As can be seen, the CMF sample exhibited an initial small amount of weight loss because of the evaporation of absorbed water on the surfaces of cellulose at a temperature below 100°C. The thermal degradation began at a temperature range of 200–300°C, indicating that the isolated CMFs was thermally stable below 200°C. The onset temperature (*T*_onset_ 90%) of microfibers was observed at 189°C with a maximum degradation temperature (*T*_max_) of 348°C. The major weight loss of CMFs was 85% at 223°C confirming cellulose degradation. The char residue was 8.1% at 600°C. The high thermal stability and low char residue of microfibers are related to the removal of non‐cellulosic compounds during alkali and bleaching treatments. Similar results were reported by Galiwango et al. (2019) who noticed that the thermal decomposition temperature of cellulose isolated from date palm biomass waste was between 200 and 250°C. According to Beroual et al. (2020), the cellulose extracted with different delignification treatments presented higher degradation temperatures in the range of 250–380°C.

### 3.2 Composite films characterization

#### 3.2.1 ATR‐FTIR analysis

**FIGURE 3:**
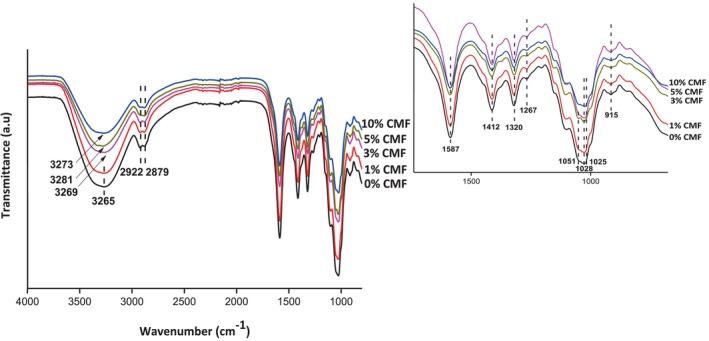
FTIR spectra of CMC/CMF composite films.

The ATR‐FTIR spectra of neat CMC and CMC‐CMF films are shown in Figure 3. The spectrum of neat CMC film (Figure 3) showed a peak at 3265 cm^−1^ assigned to the –OH stretching vibration. Peaks at 2892 cm^−1^ were related to CH and CH_2_ stretching intramolecular H bonding. These specific peaks together with the previously obtained results by XRD analysis confirmed the cellulose type I of the isolated microfibers (Rojas, 2013). The typical absorption peaks at 2922 and 2879 cm^−1^ were attributed to C‐H symmetrical stretching. The COO− stretching of the carboxylic group shows a peak at 1587 cm^−1^. In addition, the characteristic peaks at 1412 and 1320 cm^−1^ were assigned, respectively, to O–H stretching and C–H stretching vibration in the methyl group. The peak at 1267 cm^−1^ corresponds to the C–O symmetric stretching. Finally, the C–O stretching of CH_2_OCH_2_ and O–H out‐of‐plane bending show peaks at 1025 and 915 cm^−1^, respectively (Badry et al., 2020). As can be seen from ATR‐FTIR spectra of CMC/CMF films (Figure 3), the intensity of the bond assigned to –OH stretching decreased after the addition of increased concentrations of CMFs (3%, 5%, and 10%) and shifted to higher wavenumbers (3269, 3281, and 3273 cm^−1^, respectively), suggesting the formation of intermolecular hydrogen bonding between CMC biopolymer and CMFs. Furthermore, a clear peak at 1051 cm^−1^ ascribed to C–O stretching vibration was observed after the incorporation of CMFs at 5% confirming the presence of CMFs in the resulting composite films. Our results endorse that CMC and CMFs have good compatibility and interact through intermolecular hydrogen bonds without significant structural changes in CMC/CMF films.

#### 3.2.2 Surface morphology analysis

**FIGURE 4:**
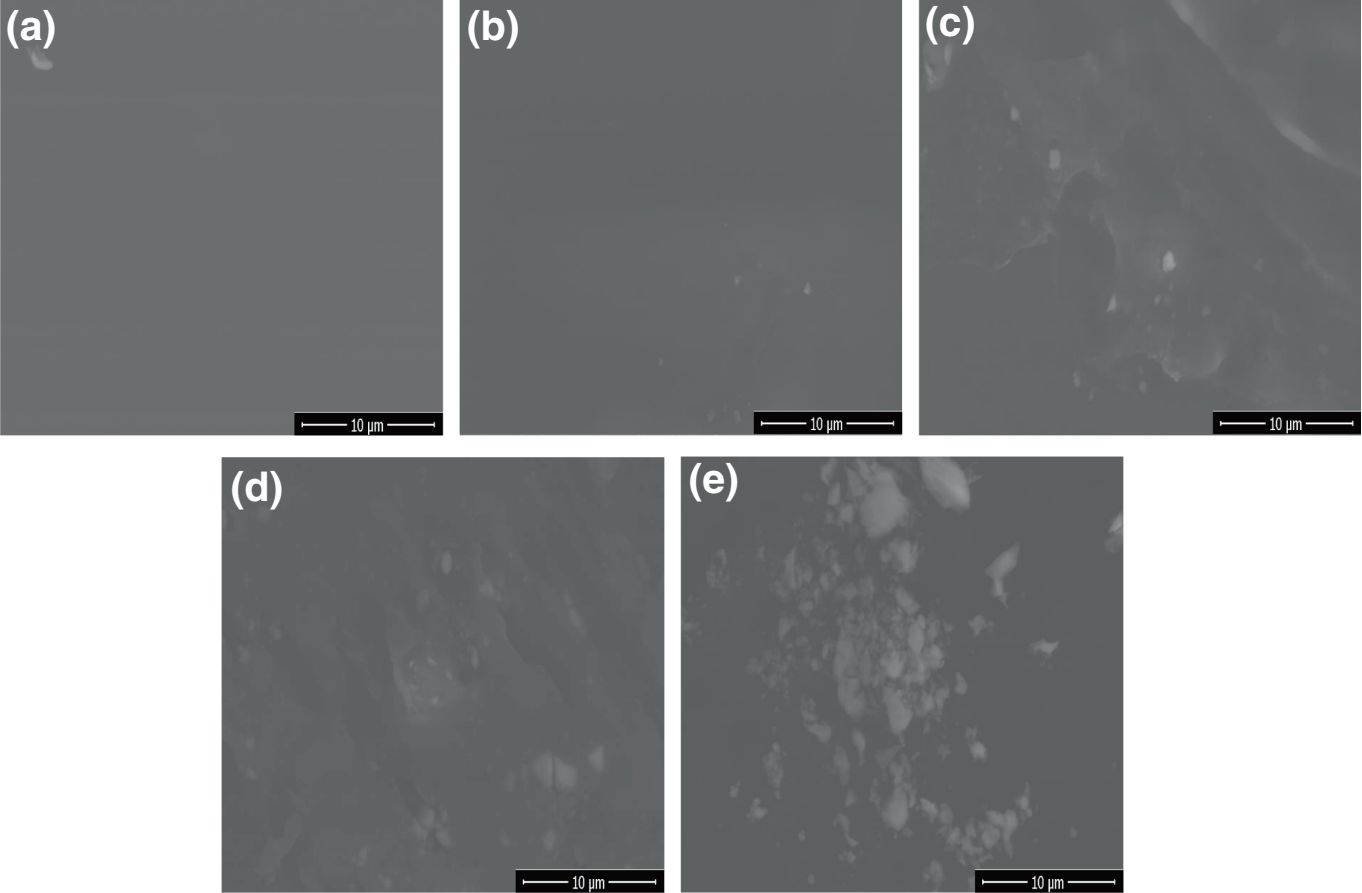
Surface morphology of the (a) neat CMC film and (b) 1% CMF, (c) 3% CMF, (d) 5% CMF, and (e) 10% CMF composite films.

As shown in Figure 4, neat CMC film has a smooth and homogeneous surface. The incorporation of CMFs in the CMC matrix resulted in the apparition of white spots on the film's surface. In composites with 1%–5% of CMFs, the spots were homogeneously and uniformly distributed in the film‐forming matrix, highlighting a good interfacial interaction between CMC and CMFs. However, composites at 10% CMFs show some agglomerates, and their uneven distribution resulted in a rough and inhomogeneous surface. Similar results were obtained by Tian et al. (2020) who noticed that CMF agglomeration resulted from CMF incorporation into starch/PVA matrix at a concentration exceeding 2% and led to the loss of the mechanical reinforcing effect of CMFs.

#### 3.2.3 Water and moisture barrier properties

The MC values of CMC/CMF composite films are reported in Table 1. The MC values were decreased significantly by incorporating 3%–10% of CMFs (*p* < .05) and this decrease exceeded 29% at 10% CMFs compared with the neat CMC film. However, no significant differences were observed between the MC values of CMC films when increasing CMF content beyond 3% (*p* > .05). The decrease in MC may be due to the reduced water trapping ability of the CMC film incorporated with CMFs which interacts with CMC, forming a denser film structure and thus decreasing moisture absorption. In agreement with our results, Teles et al. (2021) reported a significant decrease in MC of starch‐based films with increasing CMF content, which is less hygroscopic than the other film components. The determination of the solubility of films designed for food packaging is of great importance as it is related to the water resistance of the packaging and affects its biodegradability. Higher film WS values indicated lower water barrier performance. As shown in Table 1, pure CMC film displayed a pronounced hydrophilic character with a solubility value of 98.22 ± 0.65%. The WS values of CMC films decreased when increasing CMF content from 1% to 5% and the lowest solubility was reported for CMC/5% CMF films. However, the WS of CMC films remained unchanged when incorporating 10% CMFs. The increase in water barrier properties of CMC films with CMF incorporation can be attributed to the strong interaction between CMC chains and the hydroxyl groups of CMFs through intermolecular hydrogen bonds as confirmed by ATR‐FTIR analysis (Figure 3). Similar results were obtained by Liu et al. (2020) who developed biodegradable nanocomposite films from soybean polysaccharide/nano‐zinc oxide reinforced with CMFs from microcrystalline cellulose (MCC) and noticed a decrease of 37% in the WS of films after the incorporation of 10% CMFs. Conversely, they reported that a slight increase in the WS of soy protein films was observed after the addition of CMFs due to the formation of new protein–fiber interactions (Ortiz et al., 2018). Neat CMC film exhibited the highest WVP (29.19 ± 0.45 g.μm/m^2^.j. kPa) and thus the lowest moisture barrier properties due to its amorphous structure. WVP of CMC films was negatively affected by CMF content (*p* < .05). The incorporation of 5% CMFs into CMC film reduced its WVP value by more than 22% and this reduction in WVP is ascribed to the homogeneous dispersion of CMFs into the CMC matrix as revealed by SEM images (Figure 4), creating a long and tortuous path for water vapor diffusion. Likewise, Ponnusamy et al. (2022) reported a reduction in WVP by 12% after the addition of 5% CMFs in the polylactic acid (PLA) film matrix. A similar trend was observed by Guimarães et al. (2016), who attributed the greatest reduction in WVP of starch films incorporating CMFs to the good dispersion of this nanofiller into the polymeric matrix. However, when CMF loading increased in the nanocomposites to 10%, the WVP significantly increased (*p* < .05) (28.40 ± 0.65 g.μm/m^2^. day. kPa) and was similar to that of neat CMC film, mainly because of the formation of a heterogenous film structure due to nanofibers aggregation as revealed by SEM images (Figure 4).

**TABLE 1:** Mechanical and physical properties of CMC/CMF composite films.

Film	Mechanical properties				Physical properties		
	Thickness (μm)	TS (MPa)	YM (MPa)	%*E*	WVP (g.μm/m^2^. day. kPa)	MC (%)	WS (%)
0% CMFs	84.58 ± 0.59^a^	15.31 ± 1.43^a^	278.18 ± 41.06^a^	34.64 ± 3.24^a^	29.19 ± 0.45^c^	14.01 ± 0.52^b^	98.22 ± 0.65^b^
1% CMFs	85.63 ± 0.53^ab^	18.87 ± 1.52^b^	333.01 ± 37.47^a^	33.04 ± 3.77^a^	25.93 ± 0.36^b^	13.39 ± 0.08^b^	93.06 ± 3.36^ab^
3% CMFs	85.93 ± 1.04^ab^	20.69 ± 2.16^bc^	349.12 ± 48.43^a^	33.89 ± 4.42^a^	24.11 ± 1.06^ab^	10.36 ± 1.23^a^	88.62 ± 0.75^a^
5% CMFs	85.83 ± 1.03^ab^	23.58 ± 2.66^c^	496.68 ± 65.63^b^	32.90 ± 4.13^a^	22.75 ± 0.12^a^	10.01 ± 0.87^a^	86.78 ± 2.51^a^
10% CMFs	87.71 ± 0.77^b^	14.51 ± 2.10^a^	496.04 ± 71.96^b^	31.58 ± 3.05^a^	28.40 ± 0.65^c^	9.88 ± 0.71^a^	91.75 ± 1.52^ab^

*Note*: All values are expressed as means ± SD. Different letters within a column indicate significant differences (*p* < .05).

Abbreviations: CMFs, cellulose microfibers; E, elongation at break; MC, moisture content; TS, tensile strength; WS, water solubility; WVP, Water vapor permeability; YM, Young's Modulus.

#### 3.2.4 Mechanical properties

Being closely related to the ability of packaging material to resist mechanical stress during storage and distribution and to keep its structural integrity during packaging operations, the evaluation of the mechanical properties of films is essential in food packaging. The thicknesses of CMC/CMF composite films are presented in Table 1. Pure CMC films had a thickness of 84.58 μm and the incorporation of different amounts of CMFs (1%–5%) had no significant effect on their thickness (*p* > .05). In contradiction with our results, Supanakorn et al. (2021) observed that the thickness of natural rubber films decreased with increasing concentrations of CMFs from eucalyptus pulp because of the formation of condensed cellulose through hydrogen bonding. The TS, %*E*, and YM of CMC films incorporated with different CMF concentrations are shown in Table 1. An improvement in the mechanical strength of CMC films was observed after the incorporation of CMFs. The TS of neat CMC was 15.31 ± 1.43 MPa and it reached the highest value of 23.58 ± 2.66 MPa at a CMF content of 5%. The TS of CMC film increased by 23.2%, 31.2%, and 54.0% after the incorporation of 1%, 3%, and 5% CMFs, respectively. This may be ascribed to the mechanical reinforcing effect of CMFs which interacts with CMC through intermolecular hydrogen bonds as revealed by FTIR analysis (Figure 3), forming a more cohesive and compact film structure. In agreement with our results, Solikhin et al. (2018) confirmed the reinforcement effect of CMFs obtained from oil palm empty fruit bunch fibers on the mechanical strength of chitosan/PVA films and demonstrated that the interfacial interactions and the formation of a percolation structure of these nanofibers with polymer chains are crucial factors to obtain nanocomposite films with enhanced final properties. Likewise, Tian et al. (2020) observed an increase in TS of starch/PVA film by 40% after the incorporation of 2% of commercial CMFs. However, at 10% CMFs, the TS of the nanocomposite films was similar to that of neat CMC film (Table 1) and the loss of the mechanical reinforcing effect at high CMF loading may be attributed to the formation of nanofibers aggregates, resulting in a heterogeneous film structure as revealed by SEM images (Figure 4). On the other hand, increasing CMF content to 5% led to a significant increase (by 1.8 times) in the elasticity modulus and thus stiffness of the resulting CMC/CMF films when compared to the neat CMC film (*p* < .05). According to Tanpichai et al. (2014), the YM of PVA/CMF films increased by 34% when incorporating 3% CMFs and this may be due to the formation of a fibril aggregate matrix. Likewise, Boussetta et al. (2022) developed high‐density polyethylene composites reinforced with CMF isolated from raw sugar beet pulp and showed that the YM increased by 30% with increasing CMF content to 10 wt%. CMF loading had no significant effect on the %*E* of CMC/CMF composite films (*p* > .05). Our results were in contradiction with those of Liu et al. (2021) who obtained a decrease in %*E* values of gelatin‐based films after the addition of different concentrations of CMFs from MCC (5%–25%), possibly due to the limited mobility of the polymeric matrix resulting from the strong hydrogen bonds between cellulose fibers and gelatin macromolecules. Overall, the highest TS, YM, and %*E* values were obtained at 5% CMFs with 23.58 ± 2.66 MPa, 496.68 ± 65.63 MPa, and 32.90 ± 4.13%, respectively.

#### 3.2.5 Color and light barrier properties of composite films

**FIGURE 5:**
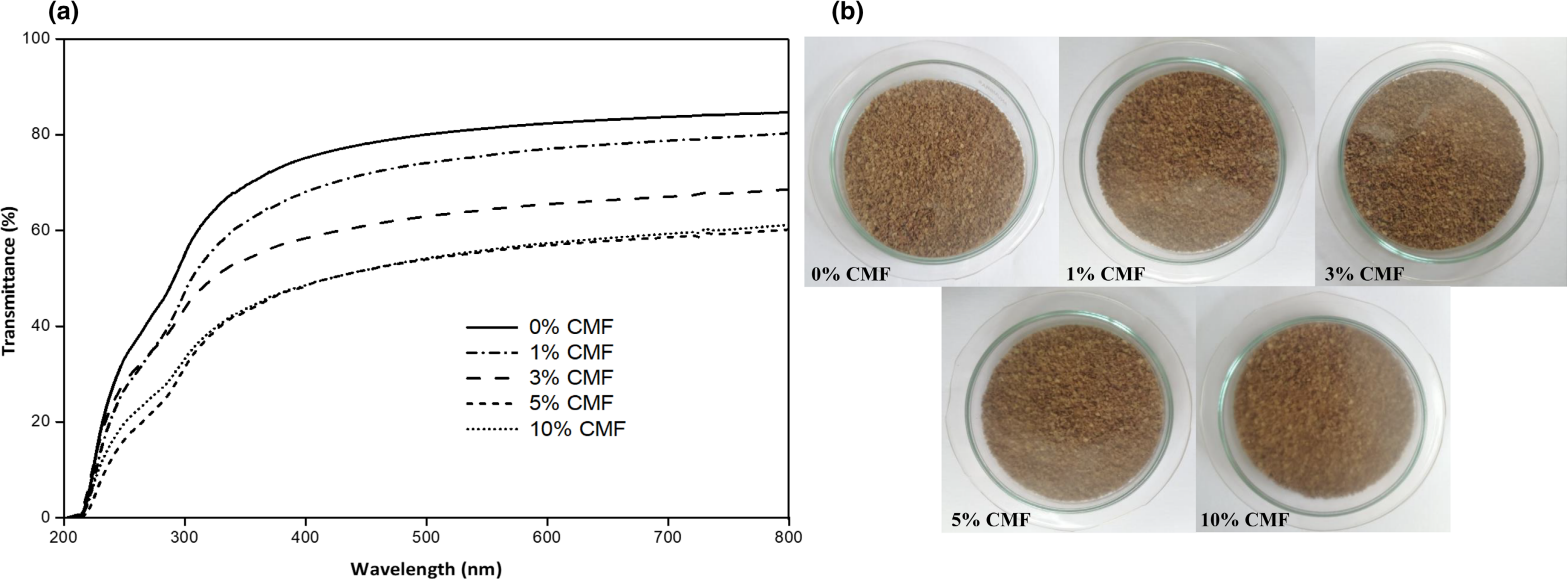
(a) UV–vis light transmittance and (b) photographs of neat CMC and CMC/CMF composite films.

The color parameters of the neat CMC and CMC/CMF composite films are shown in Table 2. A decrease in the lightness (L*) and an increase in the blueness/yellowness (b*) and the total color difference (Δ*E*) were noticed after CMF incorporation. However, increasing CMF content from 1% to 5% had no significant effect on these color parameters. Similar results were obtained by Chen et al. (2021) who developed antimicrobial starch/PVA films reinforced with commercial CMFs and noticed an increase in b* and Δ*E* values after the incorporation of these nanometric fillers in the polymeric matrix. Conversely, they reported no significant changes in L* values. The light transmittance in the wavelength range of 200–800 nm and optical photographs of CMC films incorporated with different CMF contents are shown in Figure 5. CMC/CMF‐based films exhibited lower light transmittance than neat CMC films. In general, increasing the CMF content led to a significant decrease in the transmittance of the resulting nanocomposite with 5% CMFs exhibiting the lowest light transmittance. As it can be inferred from (Figure 5a), a reduction of 47 and 30% in light transmissivity was observed for films incorporating 5% CMFs at 280 and 700 nm when compared to the neat CMC film, indicating the efficacy of the CMFs dispersed in the polymeric matrix in blocking UV and visible light path. Transparency values of neat CMC and CMC/CMF composite films are shown in Table 2. Films incorporating CMFs showed higher transparency values than neat CMC film (*p* < .05). Moreover, the observed increase in transparency values with increasing CMF content infers a significant increase in the opacity of the resulting nanocomposite films (*p* > .05). Overall, CMC films containing 5% CMFs exhibited the highest UV‐ and visible‐light‐barrier properties and can be effectively used to protect food susceptible to photo‐oxidative deterioration. A decrease in light transmission was also observed for gelatin films incorporating CMFs obtained from MCC (Liu et al., 2021). Likewise, Luo et al. (2021) also observed a slight decrease in the visible light transmittance of gelatin‐based films after the addition of commercial CMFs (0.5%–5%) because of the good dispersion and aggregation of microfibers in the different film formulations.

**TABLE 2:** Color parameters and transparency of CMC/CMF composite films.

Film	L*	a*	b*	Δ*E*	Transparency
0% CMFs	96.90 ± 0.03^c^	−0.06 ± 0.008^a^	0.61 ± 0.11^a^	3.17 ± 0.05^a^	0.93 ± 0.002^a^
1% CMFs	96.76 ± 0.03^b^	−0.06 ± 0.01^a^	0.94 ± 0.10^bc^	3.38 ± 0.06^bc^	1.37 ± 0.013^a^
3% CMFs	96.80 ± 0.01^b^	−0.03 ± 0.00^b^	0.68 ± 0.03^a^	3.27 ± 0.01^b^	2.19 ± 0.006^a^
5% CMFs	96.76 ± 0.05^b^	0.00 ± 0.01^c^	0.81 ± 0.15^ab^	3.34 ± 0.09^bc^	2.42 ± 0.025^ab^
10% CMFs	96.67 ± 0.02^a^	0.02 ± 0.01^c^	1.05 ± 0.05^c^	3.50 ± 0.04^d^	2.61 ± 0.001^b^

*Note*: All values are expressed as means ± SD. Different letters within a column indicate significant differences (*p* < .05).

Abbreviation: CMFs, cellulose microfibers.

#### 3.2.6 Thermal properties

**FIGURE 6:**
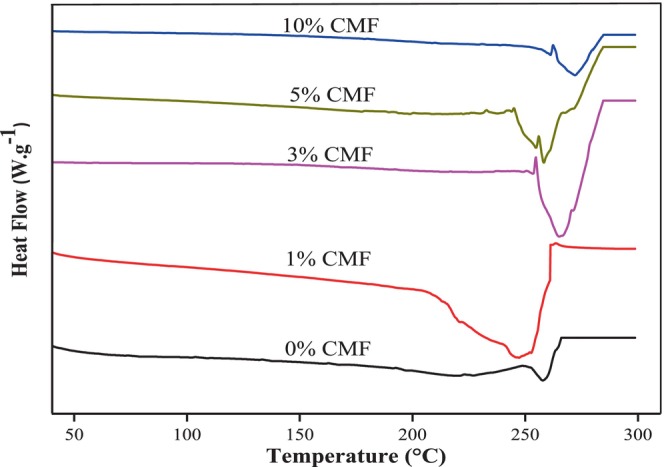
DSC thermograms of neat CMC and CMC/CMF composite films.

The DSC thermograms of different CMC/CMF composite films are shown in Figure 6. Neat CMC had a melting temperature (Tm) of 257°C. The incorporation of increasing CMF concentrations (3%, 5%, and 10% CMFs) increased Tm to, respectively, 265, 258, and 272°C. Beyond 5% CMFs, we observed splitting in the melting peaks denoting probably phase separation due to an uneven distribution as shown in SEM morphology (Figure 4). The observed improvement in thermal stability may be assigned to the formation of hydrogen bonding between CMC biopolymer and CMFs. In agreement with our results, Liu et al. (2020) noticed that soybean polysaccharide reinforced with CMFs showed enhanced thermal stability.

## 4 CONCLUSIONS

To valorize abundant and low‐cost by‐products from olive oil processing mills, CMFs were successfully extracted from olive pomace by alkali and bleaching treatments. The reinforcing capability of the obtained microfibers was evaluated using CMC as a polymer matrix by the solvent casting process. The incorporation of CMFs reduced water trapping ability and enhanced the mechanical, thermal, and water vapor barrier properties of CMC films. The highest TS, YM, and %*E* values were obtained at 5% CMFs with 23.58 ± 2.66 MPa, 496.68 ± 65.63 MPa, and 32.90 ± 4.13%, respectively. Increasing the CMF content led to a significant decrease in the transmittance of the resulting nanocomposite, with 5% CMFs exhibiting the lowest light transmittance. Overall, CMC films containing 5% CMFs exhibited the highest water‐, UV‐, and visible‐light‐barrier and mechanical properties and can be effectively used to protect food susceptible to photo‐oxidative deterioration.

## AUTHOR CONTRIBUTIONS

**Cyrine Amara:** Methodology (supporting); writing – original draft (lead). **Ayoub El Mahdi:** Data curation (supporting); methodology (supporting); visualization (supporting). **Perihan Kubra Akman:** Formal analysis (supporting); investigation (supporting). **Raouf Medimagh:** Methodology (supporting); validation (supporting); visualization (supporting). **Fatih Tornuk:** Data curation (supporting); supervision (supporting); writing – review and editing (supporting). **Khaoula Khwaldia:** Conceptualization (lead); funding acquisition (lead); methodology (lead); resources (lead); supervision (lead); validation (equal); writing – review and editing (lead).

## FUNDING INFORMATION

This work was funded by the VIPack project (TUNGER 18‐001) which is funded by the Ministry of Higher Education and Scientific Research (MHESR, Tunisia) through the Tunisian–German bilateral S & T cooperation involving science and industry (2+2 projects).

## CONFLICT OF INTEREST STATEMENT

The authors declare no conflict of interest.

## Data Availability

Data are available on request from the authors.
